# Deep Characterization of Isomerization in the Human Eye Lens Proteome by Crystallin‐Depleted Data‐Independent Acquisition

**DOI:** 10.1111/acel.70028

**Published:** 2025-05-01

**Authors:** Evan E. Hubbard, Thomas A. Shoff, Manhoi Hur, Tyler R. Lambeth, Chengwei Chen, Ethan Kung, Bruce D. Pan, Matthew K. Lui, Javian R. Linares, Lee S. Cantrell, Kevin L. Schey, Ryan R. Julian

**Affiliations:** ^1^ Department of Chemistry University of California California USA; ^2^ Department of Botany and Plant Sciences University of California Riverside California USA; ^3^ Institute of Integrative Genome Biology University of California California USA; ^4^ Chemical and Physical Biology Program Vanderbilt University Nashville Tennessee USA; ^5^ Department of Biochemistry and Mass Spectrometry Research Center Vanderbilt University Nashville Tennessee USA

**Keywords:** data‐independent acquisition, eye lens, human aging, isomerization, proteomics

## Abstract

The eye lens is a unique tissue optimized for light transmission and refraction, necessitating dissolution of all organelles in mature fiber cells. This absence of organelles prevents protein turnover and leads to the accumulation of many spontaneous modifications over time. One modification that is oft overlooked is isomerization, despite its known impact on protein structure, interference with enzymatic activity, and association with disease. Prior analysis of isomerization in the lens has been limited to a small number of targets, consisting primarily of the highly abundant crystallin proteins. Proteomic coverage can be greatly increased by first depleting the crystallins and then employing state‐of‐the‐art data‐independent acquisition (DIA) mass spectrometry (MS). However, this approach has not been combined with data analysis methods capable of identifying isomers. By so doing, we identified hundreds of previously unreported, noncrystallin Asp isomer sites. To a lesser extent, isomerization was also detected at serine and glutamic acid, consistent with previous reports of relative isomerization propensities. Interestingly, we also identify histidine isomerization sites in a select number of peptides associated with metal adduction. We further analyzed our results according to primary sequence and secondary structure to explore factors potentially influencing isomerization. Finally, we found that while isomerization percents for individual proteins are modestly accurate predictor of age, inclusion of multiple isomerized sites affords a more accurate prediction of age, which may be useful for applications in forensics.

## Graphical abstract

Long‐lived proteins can undergo spontaneous isomerization over time. By examining proteins in the eye lens, we quantified isomerization at aspartic acid, serine, histidine, and glutamic acid residues and identified both age‐dependent and structure‐dependent correlations.

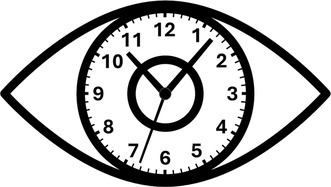

## 1 Introduction

The lens is a unique component of the human eye that focuses light into the retina for proper detection of visual stimuli. The bulk of the lens is made up of fiber cells. When these cells mature, organelles are destroyed, leaving behind a highly concentrated proteinaceous matrix that allows unimpeded transmission of light (Augusteyn and Stevens 1998). Although beneficial for sight, the absence of organelles halts most cellular functions, including protein synthesis and degradation, meaning that some proteins in the lens persist for the entire lifetime of the individual. These extremely long‐lived proteins undergo many spontaneous modifications, including deamidation, oxidation, crosslinking, truncation, and isomerization (Fujii et al. 1999; Hains and Truscott 2010; Hains and Truscott 2007; Gupta, Srivastava, and Srivastava 2006; Schey et al. 2021). All of these modifications are associated with lens aging and have the potential to contribute to disease, but isomerization is particularly challenging to comprehensively characterize.

**SCHEME 1:**
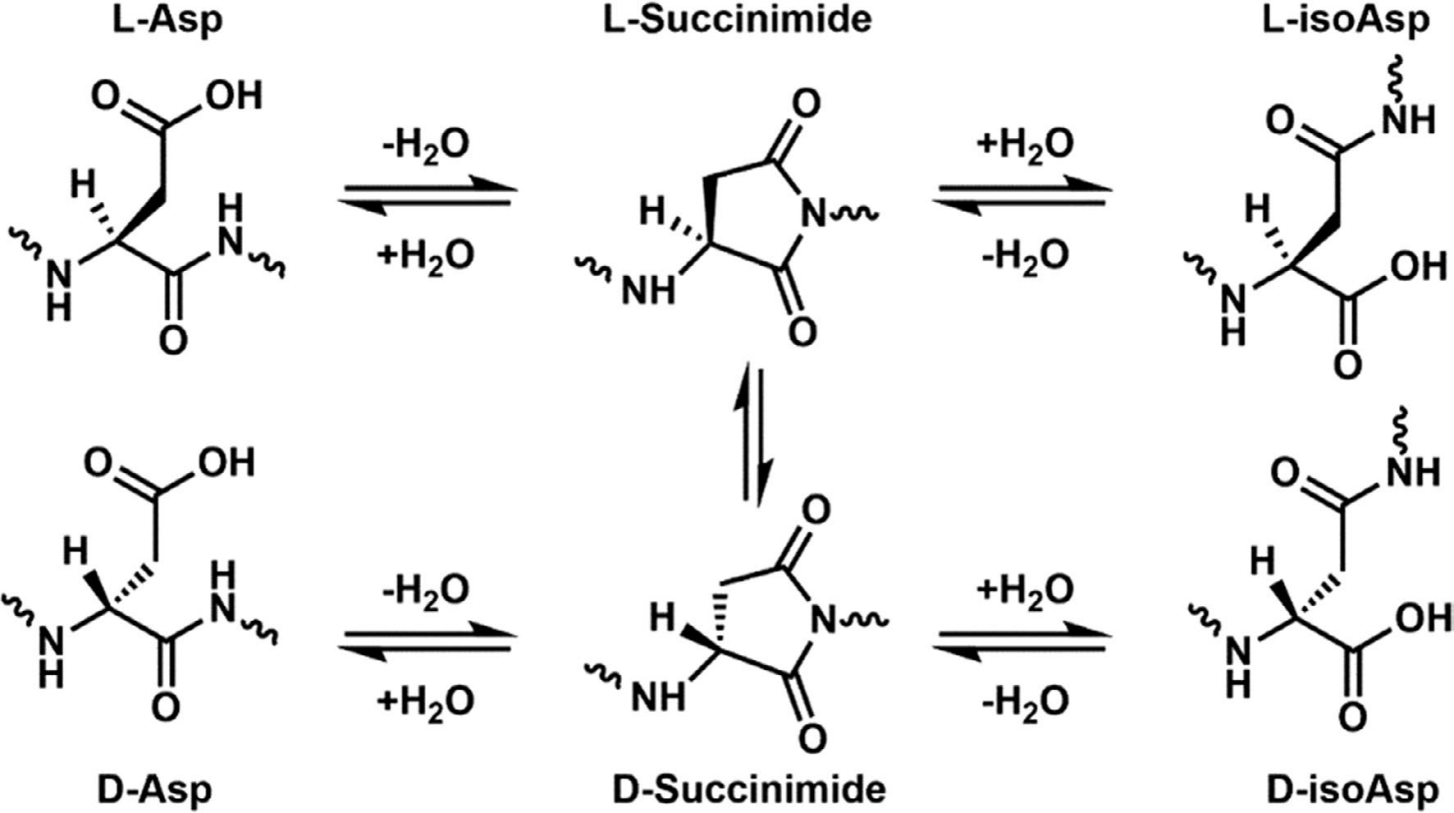
The four products of Asp isomerization produced via the succinimide intermediate.

Despite being difficult to observe, spontaneous isomerization of a single amino acid residue can lead to consequential changes in protein structure and function, including inhibition of enzymatic proteolysis and prevention of substrate recognition (Lambeth et al. 2019; Lyon et al. 2019; Silzel et al. 2022). On biological timescales, only aspartic acid (Asp) and, to a lesser extent, serine (Ser) and glutamic acid (Glu) have been found to appreciably isomerize by spontaneous chemistry (Truscott, Schey, and Friedrich 2016). Asp is most prone to isomerization due to its ability to form a succinimide ring intermediate that facilitates conversion into four possible end products (Scheme 1). Asp isomerization, largely within the crystallin proteins, has been examined in a variety of studies (Fujii et al. 1994; Tao and Julian 2014), suggesting a relationship between isomerization and lens function (Lyon et al. 2019), and revealing the importance of enzymatic repair (Lyon, Sabbah, and Julian 2018). The relationship between crystallin isomerization and cataract formation has also been explored (Fujii et al. 2016; Warmack et al. 2019; Zhu et al. 2021).

Crystallins make up the vast majority of protein in the lens, with α‐crystallins acting primarily as chaperones to prevent unchecked aggregation at the high protein concentrations needed to provide the optimal refractive index (Bloemendal et al. 2004). Collectively, roughly 90% of lens proteins are crystallins by mass, with ~40% attributed to the αA‐ and αB‐crystallin pair (Horwitz et al. 1999; Wistow 2012). The overwhelming abundance of the crystallins makes proteomic analysis of all other lens proteins particularly difficult because the dynamic range is anchored on the high side by the most abundant ions (Cantrell and Schey 2021a, 2021b). Put another way, after the crystallins have occupied most of the ion current, little is left for the analysis of the remaining proteins. Therefore, although the use of an exclusion list to avoid analysis of crystallins may help with proteome coverage, many analytes will simply not be ionized with sufficient intensity for detection. These issues are similar to those encountered in the analysis of serum, where the albumins are present in high abundance and the use of albumin depletion is common. A similar approach has recently been developed where sequential washes of the insoluble lens protein fraction with increasing concentrations of urea can be used to deplete hydrophilic proteins, including crystallins, affording greatly increased proteome coverage of hydrophobic, often membrane proteins (Cantrell and Schey 2021b; Cantrell, Gletten, and Schey 2023).

Also of importance for lens proteomics, data‐independent acquisition (DIA) has recently emerged as an effective tool for proteome‐wide analysis of aspartic acid isomerization (Hubbard et al. 2022). LC–MS proteomics methods, including DIA, can chromatographically separate peptide isomers into distinct peaks (Tao and Julian 2014). As peptides elute, DIA monitors fragments from all precursors by cycling repeatedly through a series of MS^2^ m/z windows (Zhang et al. 2020). This ensures that, for each isomerized peptide, all of its separated constituent isomer populations are consistently identified and quantified. DIA is superior to the more typical data‐dependent acquisition (DDA) method when it comes to identifying isomers. DDA utilizes single MS^2^ scans of individually selected peptides for sequence identification. If any potential isomer peak is not selected for MS^2^ analysis, its identity cannot be confirmed. The likelihood of missing isomers increases with a longer dynamic exclusion period, which is a set period of time over which a precursor ion will not be re‐examined in DDA. Longer dynamic exclusion increases proteome coverage but also decreases isomer identification, creating competing priorities that cannot be simultaneously satisfied.

In this work, we applied data analysis methods to identify and quantify isomers in crystallin‐depleted DIA‐based proteomics datasets. 223 isomerization sites were identified within this data, the vast majority of which are unreported and detected within noncrystallin proteins. These include Asp, Ser, and Glu isomerization sites, but surprisingly, histidine (His) isomerization was also detected. We use this whole‐proteome picture of isomerization to better understand what dictates isomerization rates in tissues with extremely low turnover. We also report the accurate prediction of lens age by merging information from many isomerized peptides simultaneously.

## 2 Experimental

### 2.1 Analysis of Lens DIA Data Using Skyline

Data from a previously published human lens proteome study were analyzed in this study (Cantrell, Gletten, and Schey 2023). Thermo .raw files were downloaded from ProteomeXchange (dataset entry PXD033722) via an FTP link ftp://massive.ucsd.edu/v04/MSV000089427/ using FileZilla (version 3.65.0). These files were converted to mzML with ProteoWizard MSConvert using the generic defaults preset, “SIM as spectra” selected, vendor level peak picking, and demultiplexing (Adusumilli and Mallick 2017). For each lens region, mzML files were imported into Skyline (version 23.1.1) as three separate Skyline documents (MacLean et al. 2010). The spectral library for each document was built in Skyline, starting from “DIA raw” with a 0.95 cutoff score, ambiguous matches included, and a DIA workflow. The canonical human FASTA obtained from Uniprot was used for library generation, with 1% FDR and decoys generated by shuffling the sequence of every target. Search parameters were as follows: deamidation of asparagine and glutamine were included as a possible PTMs, 2+ and 3+ precursor charges were allowed, up to one missed cleavage was allowed, five product ions were picked (with a three‐product minimum), and a 10 ppm mass tolerance was chosen for MS^1^ and MS^2^ filtering.

DIA‐Umpire was used during document generation to construct chromatogram spectral libraries within Skyline (Tsou et al. 2015). MS Amanda was selected as the search engine, with 10 ppm mass tolerances for both MS^1^ and MS^2^, and 2+/3+ allowed as considered precursor charges (Dorfer et al. 2014).

### 2.2 Searching for Isomerization in the Lens Proteome

The presence of isomerization in Asp‐containing peptides was determined manually, within the inner‐nucleus sample cohort, using chromatographic information. We searched for isomers in Skyline, filtering for only Asp‐containing peptides using the document grid. To search for Ser and His isomers, peptides were filtered to always include either residue; then Asp was excluded as an allowable residue. To confirm Asp, His, or Ser isomers, chromatograms were visually assessed for the presence of multiple peaks. The minimum qualifications for acceptance as a potential isomer peak were the presence of precursor signal, signal for at least three fragments, and all ions forming an approximately Gaussian distribution centered on a single retention time.

### 2.3 Automated Quantification of Isomerization

% Isomerization was calculated using the area values for each isomer peak within Skyline chromatograms. The ratio of the total noncanonical isomer peak area and the total area of all peaks was expressed as a percentage (Equation 1). Synthetic peptide standards were impractical for distinguishing the canonical L‐Asp form from the other isomers in hundreds of analytes. Instead, the change in relative peak area was used for identification. If the area for one peak decreased relative to all others from younger to older lens samples, then it was considered the L‐Asp form, and all other peaks were the accumulating, noncanonical Asp forms. If there was no relative change with age, then the peptide was not considered for quantification.

(1) $$\%\mathrm{Iso}.=\frac{\sum \text{Isomer Peak Area}}{\sum \text{Total Peak Area}}$$

Identifying isomers in each chromatogram requires the simultaneous provision of individual total peak areas for multiple detected RT peaks (or isomers). Since Skyline offers the total peak area of all precursors and product ions within a peak boundary based solely on user‐selected boundaries in chromatograms, we added an automated algorithm for the simultaneous detection and total peak area extraction of multiple RT peak boundaries into Skyline version 21.2 (MacLean et al. 2010). To maintain compatibility with the quantification tool, all Skyline files were reverted to this version using the “Share” tool in Skyline.

The algorithm requires the user to select a reference chromatogram where isomers are present. The isomers in this reference chromatogram are then used to identify isomers in other chromatograms. Additionally, the parent isomer peak with the highest intensity in the reference chromatogram, known as the base RT peak, is used to align the chromatograms. Since chromatograms often experience RT shift issues due to factors such as matrix effects, long gradient times in experiments, software bugs, etc., the base RT peak from the reference chromatogram is used to realign them, ensuring accurate isomer detection. However, in some cases, the base RT peak of the parent isomer may not have the highest abundance among the chromatograms. To address this, the algorithm allows the user to manually input the apex RT value of the parent peak along with RT margin windows.

Next, the algorithm detects RT peaks and establishes peak boundaries in all chromatograms, including the reference chromatogram, using the peak detection algorithm. To detect RT peaks and their boundaries, the data series of a precursor or product ion in a peptide is required. Alternatively, the sum or average of precursors or products can be used. To reduce noise and enhance RT peak detection, this process uses smoothing methods such as moving average smoothing and Savitzky–Golay smoothing (Yang, He, and Yu 2009). By using the RT and margin windows of the RT peaks observed in the reference chromatogram, the algorithm identifies RT peaks with similar RT and abundance patterns in other chromatograms to verify whether they are isomer peaks. For this verification, the Bray–Curtis Similarity (BCS) algorithm is employed, as even though chromatograms are realigned, the alignment may not be perfect (Bray and Curtis 1957). This BCS algorithm evaluates the similarity between two RT peaks within a boundary across different chromatograms by comparing their start, middle, and end RT values.

$$\text{Bray}-\text{Curtis Similarity}\;\left(\%\right)=\left(1-\frac{\sum_{i=1}^{n}\left|x_{i}-y_{i}\right|}{\sum_{i=1}^{n}\left(x_{i}+y_{i}\right)}\;\right)\times 100$$

In the BCS algorithm, x_*i*_ represents the retention time at position _*i*_ in the RT peak within a chromatogram, while y_*i*_ represents the corresponding retention time in the isomer peak within the reference chromatogram. The variable *n* denotes the number of elements in these RT peaks. The similarity score ranges from 0% to 100%, with 100% indicating identical similarity and 0% indicating complete dissimilarity. The cutoff similarity score is set at 99.0%, and any score lower than that will be considered dissimilar. By applying this algorithm, the degree of similarity between the RT peak patterns in the reference chromatogram and those in other chromatograms is assessed, helping to confirm whether the detected RT peaks are indeed isomer peaks present in the reference chromatogram, even in cases of misalignment. The total peak area for all RT peaks within the verified RT peak boundaries across all chromatograms is then calculated by calling the custom ChangePeakBounds function, which adjusts the RT peak boundary based on the isomer's start and end RT and utilizes the functionality already implemented in Skyline.

Finally, the algorithm generates three report files: a data report table as a text file, a visual report as an HTML file, and an RT alignment checker report as an HTML file, all automatically saved in a local folder. The data report table includes the total area of the detected isomers, along with the RT, abundance, and total area of the peaks found in each individual chromatogram. The visual report plots the peaks detected in each chromatogram, providing a clear visual discrimination of the results. Lastly, the RT alignment checker report displays the peaks in chromatograms that have been realigned by the base RT peak, helping to verify the accuracy of isomer detection.

After confirming accurate peak determination from the visual report, result files were imported into Excel and percent isomerization was calculated from reported peak areas. If a clear, significant interference signal for any precursor or fragment ion was observed overlapping with any peaks, that ion was completely excluded from quantification to minimize error.

### 2.4 Sequence and Structure Analysis

PDB files for each protein were analyzed in Maestro (version 13.4). The secondary structure of the Asp of interest was classified as being alpha helical, beta sheet, or nonregular where the structures have nonrepeating backbone torsion angles (Qi and Xiao 2002). Isomers with multiple, nonsequential Asp sites were excluded from sequence and structure analyses due to the inability to confirm which Asp is isomerized. Two‐sample t‐tests assuming unequal variance were performed in OriginPro 2024 (10.1.0.178) to evaluate statistical differences between sequence motifs or structures.

### 2.5 Identifying Histidine Isomerization Within Crystallin Peptide Chromatograms

L‐Asp/D‐Asp, L‐Ser/D‐Ser, and L‐His/D‐His synthetic standards of the crystallin peptides IQTGLDATHAER and HFSPEDLTVK were synthesized based on a previously published solid‐phase peptide synthesis procedure (Hood et al. 2008). Mixtures of synthetic standards were analyzed by LC–MS using an Agilent 1100 HPLC system and Thermo Fisher Scientific LTQ mass spectrometer. Relative elution orders of these standards were used to identify the peptide isomers observed in lens tissue, including D‐His peaks found within the chromatograms.

### 2.6 Prediction of Age by Combined Linear Regression

% isomerization values at five ages (18, 34, 49, 63, and 74 years old) were quantified for 100 Asp‐containing peptides. From these, a selection of 14 peptides was used for age prediction by only including peptides with no resolution issues (i.e., all Asp isomers are separated) and a highly linear relationship between age and isomerization. Average isomerization for these peptides was calculated for all ages, and linear regression was used to predict age from these averages.

### 2.7 Isomeric Peptide Synthesis and Confirmation of Co‐Elution by LC–MS

VLPWADR and GDLGIEIPAEK were selected for peptide synthesis based on clear isomerization in the lens but less than four peaks, implying that at least two isomer peaks are unresolved. All four Asp isomers of these peptides were synthesized using a standard solid‐phase peptide synthesis approach (Hood et al. 2008). The synthesized peptides were then lyophilized to dryness, dissolved in 1 mL of H_2_O, and frozen at −20°C prior to use. The sequence was confirmed by nESI of 10 μM peptide in H_2_O with 0.1% formic acid to verify the exact mass and fragmentation patterns consistent with those of the desired peptide.

Monophasic C18 analytical columns were prepared by pulling 100 μm diameter fused silica columns with a P‐2000 laser tip puller (Sutter Instrument Co., Novato, CA) and packing to a length of 20 cm with Reprosil‐Pur 120 C18‐AQ 1.9 μm resin. All samples were analyzed using a Thermo UltiMate 3000 RSLC interfaced to a Thermo Fisher Scientific Orbitrap Fusion Lumos Tribrid Mass Spectrometer; 12.5 pg of individual peptides and mixtures of all isomers were separately analyzed. Samples were eluted using 0.1% formic acid in water as mobile phase A and 0.1% formic acid in 80% acetonitrile as mobile phase B. Samples were desalted by loading onto an Acclaim PepMap 100 C18 3 μm 75 μm × 2 cm trap cartridge at 1% B for 2.2 min. Peptides were then separated with a 90‐min LC gradient consisting of 1% B for 3 min, 1%–7% B in 7 min, 7%–15% B in 20 min, 15%–50% B in 40 min, 50%–98% B in 5 min, 98% B for 10 min, and a wash of 1% B for 5 min. The analytical column was then equilibrated at 1% B for 100 min. The instrument was operated in parallel reaction monitoring mode with the only targets consisting of multiple charge states of the synthetic peptides. MS1 scans were collected with a resolution of 120,000 and a scan range of 350–1500 m/z. MS2 scans used HCD with 33% collision energy, an automatically determined AGC target, a resolution of 15,000, a max injection time of 22 ms, an isolation window of 1.6 m/z, and a scan range of 350–2000 m/z.

## 3 Results and Discussion

**FIGURE 1:**
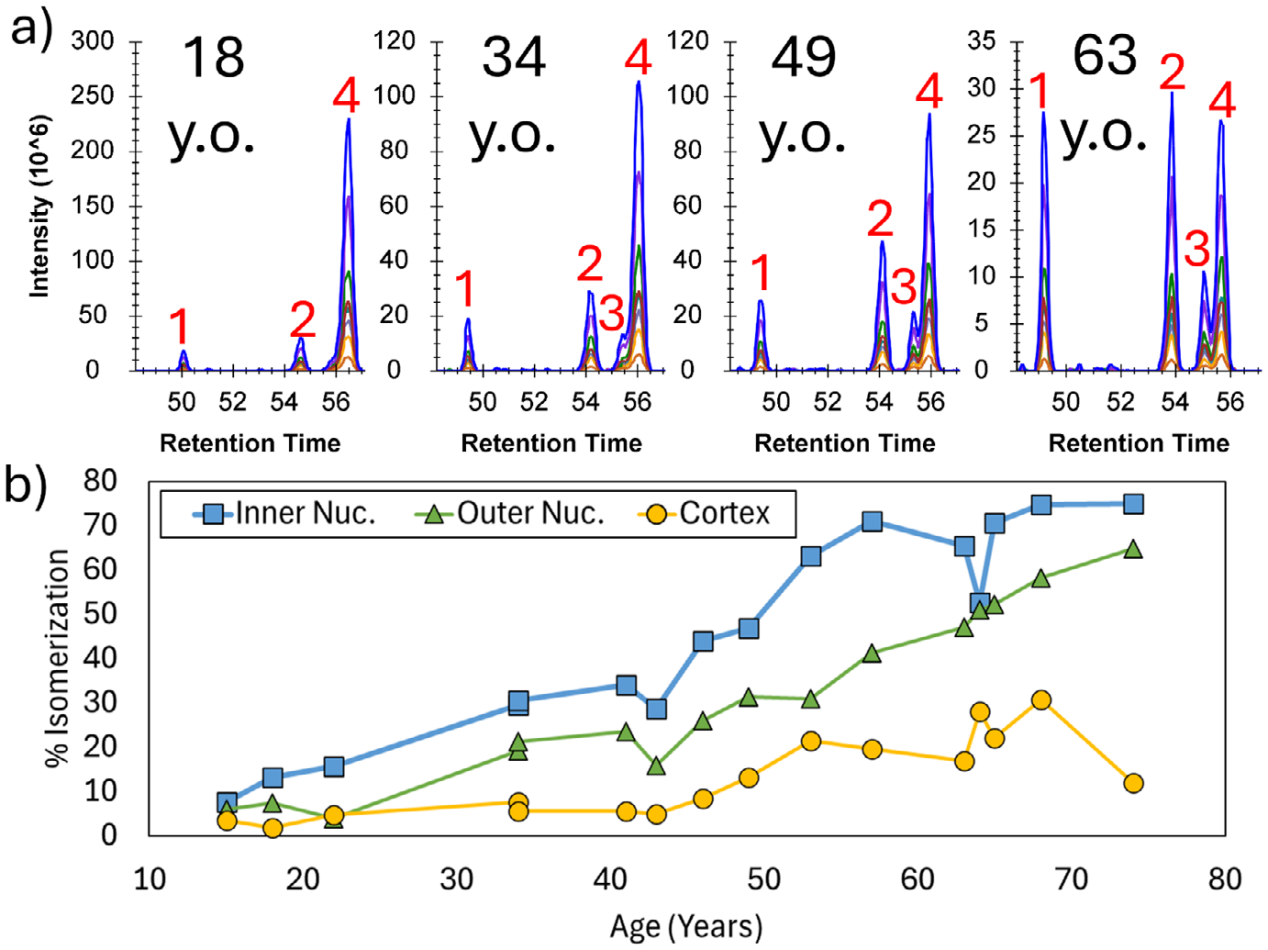
Isomerization in the peptide LGELAGPEDALAR from the protein BFSP1. (a) Chromatograms for the inner nucleus from a distribution of lens ages, each ~15 years older than the previous tissue sample. (b) % Isomerization of the peptide quantified at each age and in each region of the lens.

The primary advantage of DIA experiments is that, in theory, fragmentation data are collected for all peptides, including any isomers that might be present. However, the primary disadvantage is that this fragmentation information is not coupled to specific precursor ions. Current automated data analysis protocols for DIA data are not designed to identify isomers, requiring manual intervention as described in the experimental section. Examination of DIA data from the inner nucleus for Asp isomers yielded 220 matches (see Table S1). Illustrative results for the peptide LGELAGPEDALAR from filensin (BFSP1) are shown in Figure 1a. The presence of four chromatographic peaks tracking for the same precursor mass and fragment ions are signatures for Asp isomerization. Furthermore, the relative abundances of the peaks follow a pattern with increasing age. To clarify, peak 4 is dominant in the youngest lens, while the abundance of the other three peaks steadily increases with age for the remaining samples. Given that L‐Asp is the canonical form originally present in all peptides and that isomerization leads to the conversion of L‐Asp into the other 3 isomeric forms, the trends in Figure 1a reveal that peak 4 corresponds to L‐Asp. Unfortunately, the retention order for Asp isomers is not consistent from one peptide to another (Lyon, Sabbah, and Julian 2018), meaning that the identities of the remaining isomers cannot be easily obtained. However, the total extent of isomerization into all 3 noncanonical forms can be calculated (see Equation 1).

Shown in Figure 1b is a plot of isomerization of LGELAGPEDALAR in 3 different regions of the lens for 16 samples ranging in age from 15 to 74 y.o. Isomerization exhibits an upward trend with increasing age in all three regions of the lens, with the inner nucleus reaching the highest levels, followed by the outer nucleus, and then cortex. Given that the human lens continues to grow throughout life, with new fiber cells populating the periphery of the cortex, these trends are consistent with the expected ages for each lens region (Fujii et al. 2016). The addition of trend lines reveals that there is significant variation in the extent of isomerization between different individuals, which is not unexpected. The plot in Figure 1b is representative of the trends that were observed for the remaining peptides in Table S1. Interestingly, the data may suggest that some tissue exchange occurred between the inner nucleus and cortex for the 63‐year‐old sample as uncharacteristic opposing blips are noticeable in the data (i.e., a downward dip in isomerization for the inner nucleus concurrent with an upward tic in the cortex).

**FIGURE 2:**
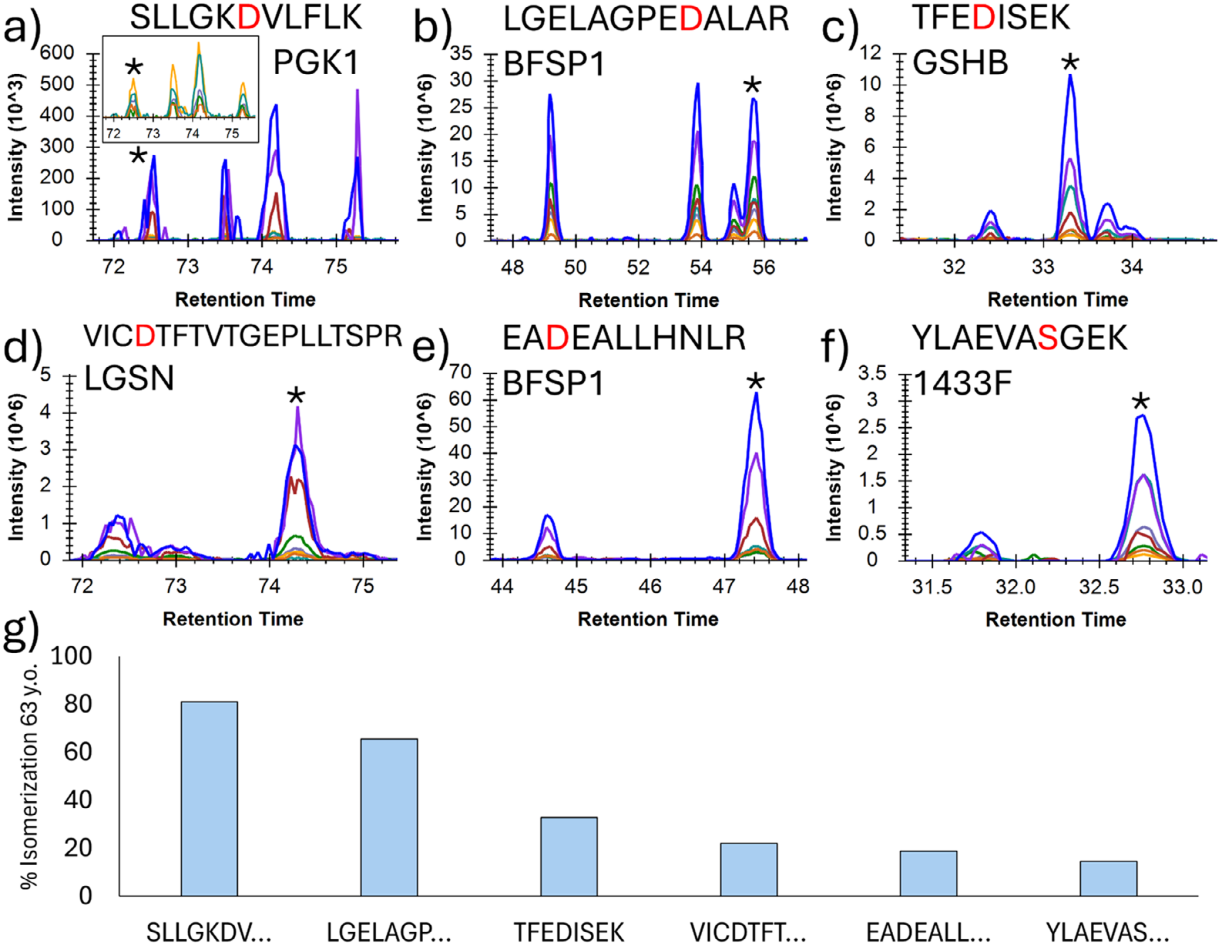
Six noncrystallin isomers identified using chromatographic information in 63 y.o., inner nucleus lens proteome. (a–e) Asp isomers, which vary widely in amounts of isomerization and retention order of isomer peaks. Asterisk denotes assumed canonical, L‐Asp peak. 2a includes insert of fragment‐only chromatogram, which was used for quantitation. (f) A serine isomer. Serine only takes on two distinct structures, producing two chromatographic peaks rather than four. (g) % Isomerization calculated from each chromatogram.

Chromatograms obtained for six additional peptides from a single 63‐year‐old lens are shown in Figure 2a–f, with the % isomerization for each peptide being quantified in Figure 2g. Although the peptides all derive from a single lens, and two peptides derive from a single protein (2b and 2e), the extent of isomerization varies from ~80% to less than 20%. Asp is the most likely site of isomerization for five of the peptides, with Ser being the likely site for the sixth peptide. Ser is the second fastest isomerizing residue and is only capable of chiral inversion to produce two isomers rather than the four observed for Asp (Demarchi et al. 2013). It is also clear by examination of elution profiles in Figure 2a–e that neither the elution orders nor the relative spacing between Asp isomers are reproducible for peptides of differing sequence. Furthermore, although all four Asp isomers are frequently resolved, there are also instances where this does not occur, as only two isomers are resolved in Figure 2e. Indeed, the extent of isomerization for this peptide may be greater than that reported in Figure 2g because some isomers may be coeluting with the canonical form. If an isomeric form coelutes with the canonical peptide, the intensity of the canonical form may not appear to change significantly over time because it is being replaced by the isomer. In instances where the number of resolved Asp isomers is less than four, the % isomerization should be considered a lower limit.

To confirm that coelution is the cause of fewer than four observable chromatographic peaks, we synthesized all isomers of the peptides VLPWADR and GDLGIEIPAEK. Each of these peptides was used as an example since they showed high isomerization in the lens but incomplete separation, with only three peaks in both cases. Figure S1a–e and S1f–j show the elution profile for each individual isomer and the mixture of all isomers for GDLGIEIPAEK and VLPWADR, respectively. In both of these cases, it is clear that the “missing” isomeric peak is unresolved due to coelution with a different isomeric peak. This result affirms that in cases where fewer than the expected numbers of isomer peaks are observed, the likely cause is coelution.

**FIGURE 3:**
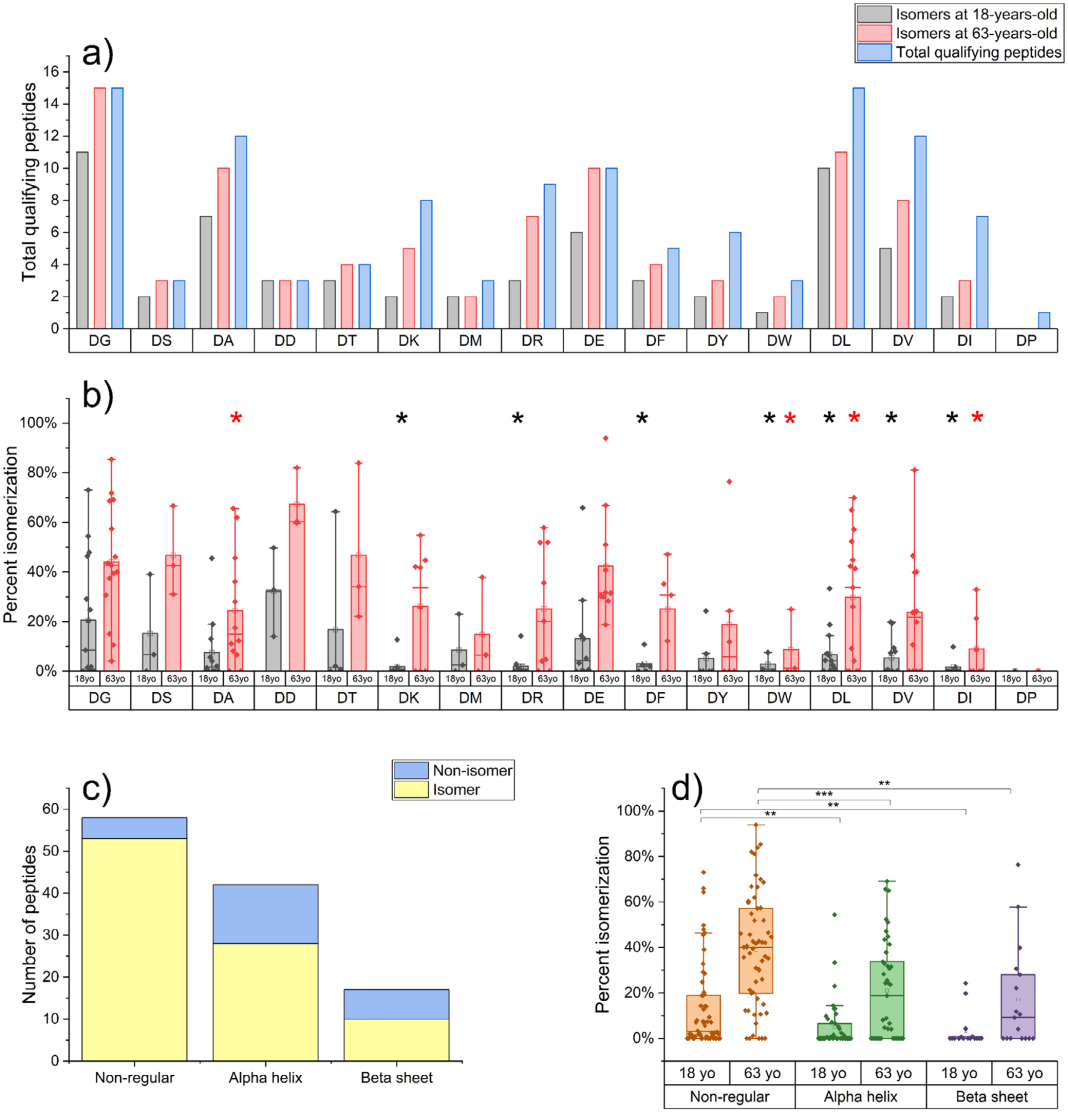
(a) Number of Asp isomers at 18‐years‐old (black) and 63‐years‐old (red) alongside the total number of qualifying peptides (blue) for each Asp residue pair (b) %isomerization binned by residue C‐terminal to Asp for both 18‐year‐old (black) and 63‐year‐old (red) lens samples. Motifs with * are statistically different from DG (*p* < 0.05) with colors corresponding to sample age. (c) Number of isomers and nonisomers for alpha helices, beta sheets, and nonregular secondary structures (d) %isomerization binned by secondary structure, including nonregular (orange), alpha helix (green) and beta sheet (purple) at 18‐years‐old and 63‐years‐old. ** corresponds to *p* < 0.01, *** corresponds to *p* < 0.001.

Having identified many Asp isomerization sites, we next compared the extent of isomerization to a variety of protein characteristics to explore potential factors that could influence the isomerization rate. Previous work on Asn deamidation (a process also occurring via a succinimide intermediate) found the identity of the amino acid C‐terminal to the site of deamidation to be important (Robinson and Robinson 2001). In Figure 3, the relationship between the identity of the residue C‐terminal to Asp is compared to isomerization in several contexts. First, Figure 3a shows the number of detected isomers in a young and old sample, 18 years old and 63 years old respectively, relative to the total number of peptides for each residue pair. To reliably determine the specific Asp isomerizing, only single‐Asp‐containing peptides were utilized, except for DD sites, which have previously been shown to isomerize rapidly (Lyon, Sabbah, and Julian 2017). Collectively, these constraints greatly reduce the number of peptides under consideration. Nonisomerized peptides were also stringently assigned, requiring a single chromatographic peak, high signal, and clear absence of isomeric peaks. DG isomers, expected to isomerize quickly due to lack of steric hindrance (Oliyai and Borchardt 1994), represent the highest number of isomers, and all DG peptides show some isomerization by 63 years old. This is not observed for sites predicted to isomerize slowly due to steric bulk, such as DL, DV, and DI. However, sites such as DE and DR show a high propensity to isomerize despite their sterically bulky side chains. Furthermore, the number of isomers found at each site does not have a straightforward relationship with sequence. To determine whether the extent of isomerization is influenced by the steric bulk of the C‐terminal amino acid, Figure 3b shows %isomerization plotted for each motif at 18 years old and 63 years old. At 18 years old, multiple sterically bulky sites, such as DF, DL, and DV, show significantly lower isomerization when compared to DG sites. DK and DR also do not isomerize as much by this age, but analysis of these peptides is complicated by the inefficiency of trypsinization adjacent to isomerized Asp (Silzel et al. 2022). At 63 years old, many of these statistical differences are no longer observed, and there are no clear trends in %isomerization. Motifs with low steric bulk, such as DS, DA, and DT, do not show statistical differences from DG at either age. Overall, the steric bulk of the C‐terminal amino acid influences isomerization at earlier time points, but this effect is diminished as proteins continue to age.

The observation that peptides reach a wide variety of %isomerization values in the lens implies that other factors must prevent most sites from reaching equilibrium. Another characteristic of proteins that may influence isomerization is the formation of secondary structures. Rates of deamidation in proteins were shown to be lower in alpha helical and beta sheet structures, owing to increased backbone rigidity limiting succinimide ring formation (Kosky et al. 1999). To see whether these same trends are observed in lens proteins, we identified the secondary structure of all Asp‐containing peptides as alpha helical, beta sheet, or nonregular. Nonregular structures lack consistent backbone torsion angles and tend to be more flexible (Qi and Xiao 2002). Figure 3c shows that isomers occur more frequently in nonregular regions than in alpha helices or beta sheets, and peptides that do not isomerize at all are primarily found in structured regions. To see whether the extent of isomerization is impacted by secondary structure, Figure 3d shows %isomerization binned by nonregular, alpha helix, or beta sheet for both 18‐year‐old and 63‐year‐old lens samples. At both ages, there is a clear, statistically significant reduction in isomerization in structured regions, particularly strong in beta sheets, compared to nonregular regions. Taken together, Figure 3c,d implies that the rigid backbone found in alpha helices or beta sheets has the potential to slow or even fully stop succinimide ring formation and subsequent Asp isomerization from occurring, even after extremely long periods of time. Formation of beta sheet isomers may require partial or full denaturation of the sheet structure. In 18‐year‐old lens samples, it is more likely that these structures have survived intact and slowed isomerization. However, proteins in highly aged samples are more likely to become misfolded (Roskamp et al. 2020) or destabilized by hydrolysis (Ray 2015; Lyons, Jamie, and Truscott 2014). This could explain why the range in %isomerization for beta sheet isomers is significantly larger at 63 years old. However, even with extensive aging, it is clear that some persistence of structure has slowed the overall isomerization rate. Taken as a whole, the extremely long progression of isomerization in the lens implicates backbone rigidity rather than steric hindrance as a more important determinant of isomerization rate.

**FIGURE 4:**
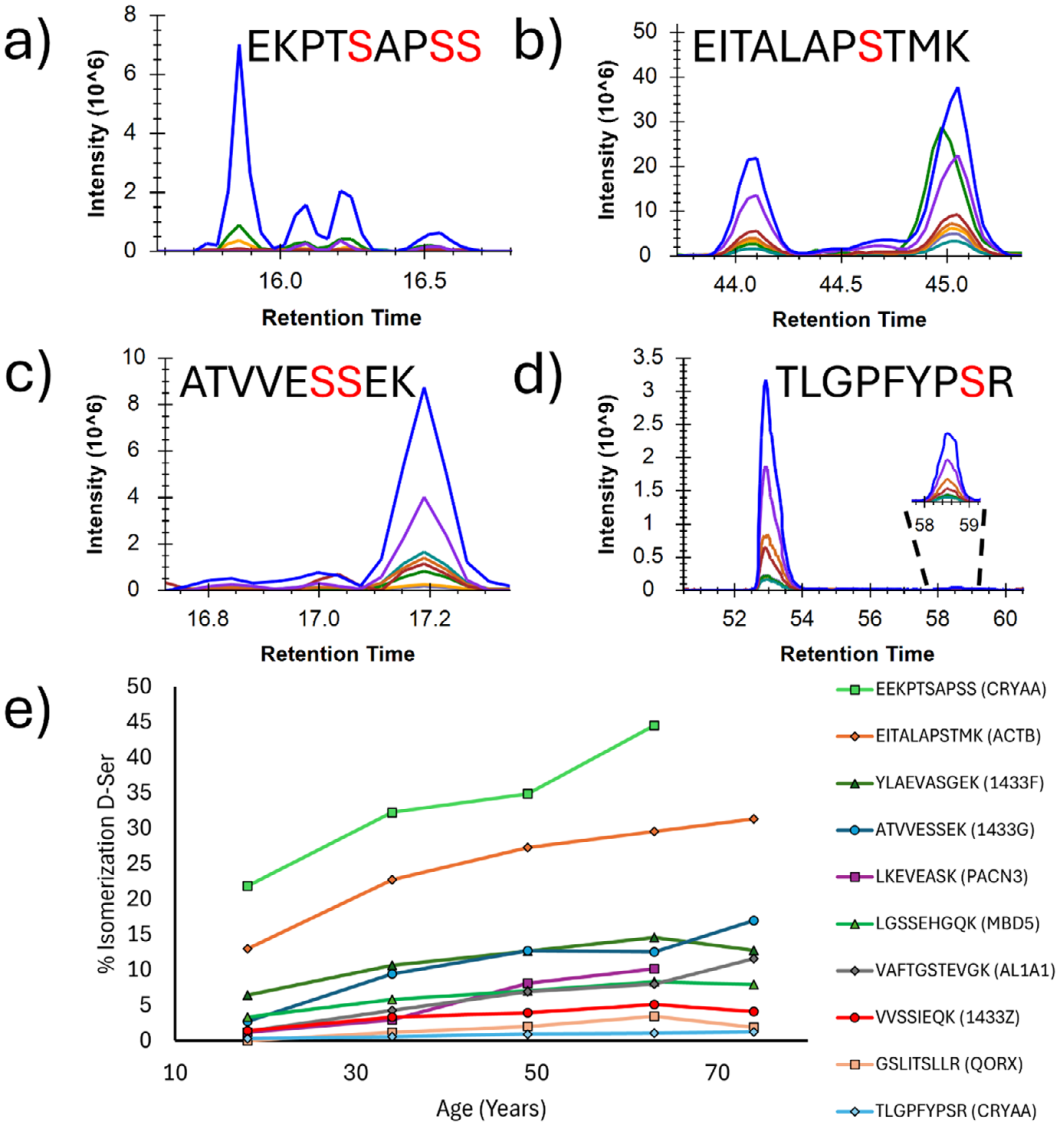
(a–d) Chromatograms of some serine isomerization sites within 63 y.o. lens tissue. (e) Ten serine isomers quantified in the inner nucleus at five timepoints from 18 to 74 y.o. Two 74 y.o timepoints are excluded due to loss of peptide signal with age. Protein identifiers are indicated in parentheses.

In addition to aspartic acid, serine isomer sites were also found within the lens proteome. Ser amino acid isomerization into the enantiomeric, D‐Ser form is known to be a biologically relevant modification. Free D‐Ser is produced in mammals via enzymatically driven processes (Nishikawa 2011). However, Ser is also capable of in‐chain, spontaneous isomerization in long‐lived proteins, much like aspartic acid (Shapira and Chou 1987). This isomerization is thought to proceed through deprotonation of the α‐hydrogen, which may be facilitated by the hydroxyl sidechain (Takahashi, Kobayashi, and Oda 2010). Ser isomerization has been examined in tryptic peptides from crystallins using bottom‐up proteomics, in model peptides, and in acid‐hydrolyzed lens proteins (Lyon et al. 2019; Hood et al. 2008; Hooi and Truscott 2011; Hooi, Raftery, and Truscott 2013). In our DIA data, 10 peptides with unambiguous isomerization at Ser were identified, and the extent of isomerization is plotted for each peptide in Figure 4. Most peptides are < 15% isomerized across all ages and samples, including the inner nucleus of a 74 y.o. However, two peptides are more extensively isomerized. One of these, EEKPTSAPSS from αA‐crystallin (CRYAA), has more Ser residues than any other Ser peptide quantified, one of which is the protein C‐terminus, all intrinsically disordered and with high solvent accessibility (Kaiser et al. 2019). These unique properties might explain its extensive isomerization. The other peptide, EITALAPSTMK from actin (ACTB/ACTG), is more difficult to rationalize. The isomerized Ser of this peptide is found in a highly solvent‐accessible region, where α‐H deprotonation is most likely (Galkin et al. 2008). However, other less‐isomerized D‐Ser sites are also exposed, and there is no clear trend between accessibility and Ser isomerization rate among the observed sites.

Alongside serine and aspartic acid, glutamic acid (Glu) is also known to isomerize via a similar mechanism to Asp (Figure S2a). Spontaneously, L‐Glu can form a glutaramide intermediate that can open via ring hydrolysis to form L‐isoGlu. This ring can also undergo chiral inversion, leading to the formation of D‐Glu and D‐isoGlu (Riggs et al. 2019). Searching Glu‐containing peptides in the lens DIA data revealed a total of 10 Glu isomers, and a series of example chromatograms are shown in Figure S2b–e. Isomerization for these peptides ranges from as low as 1% to as high as 77%, with an average of ~24% at 63 years old (Table S1). Interestingly, the peptides that reached the highest isomerization values had at least one site where Gly is C‐terminal to Glu, suggesting that a lack of steric hindrance may accelerate Glu isomerization similarly to what is observed for Asp. AEGAATEEEGTPK, the peptide that reaches the highest Glu isomerization, has two different EG sites as well as multiple E residues sequentially, both of which are known to drive isomerization in Asp (Lyon, Sabbah, and Julian 2017; Oliyai and Borchardt 1994). Although a relatively low sample size limits potential conclusions, it is a reasonable hypothesis that Glu follows the same trends related to steric hindrance as Asp in the lens.

**FIGURE 5:**
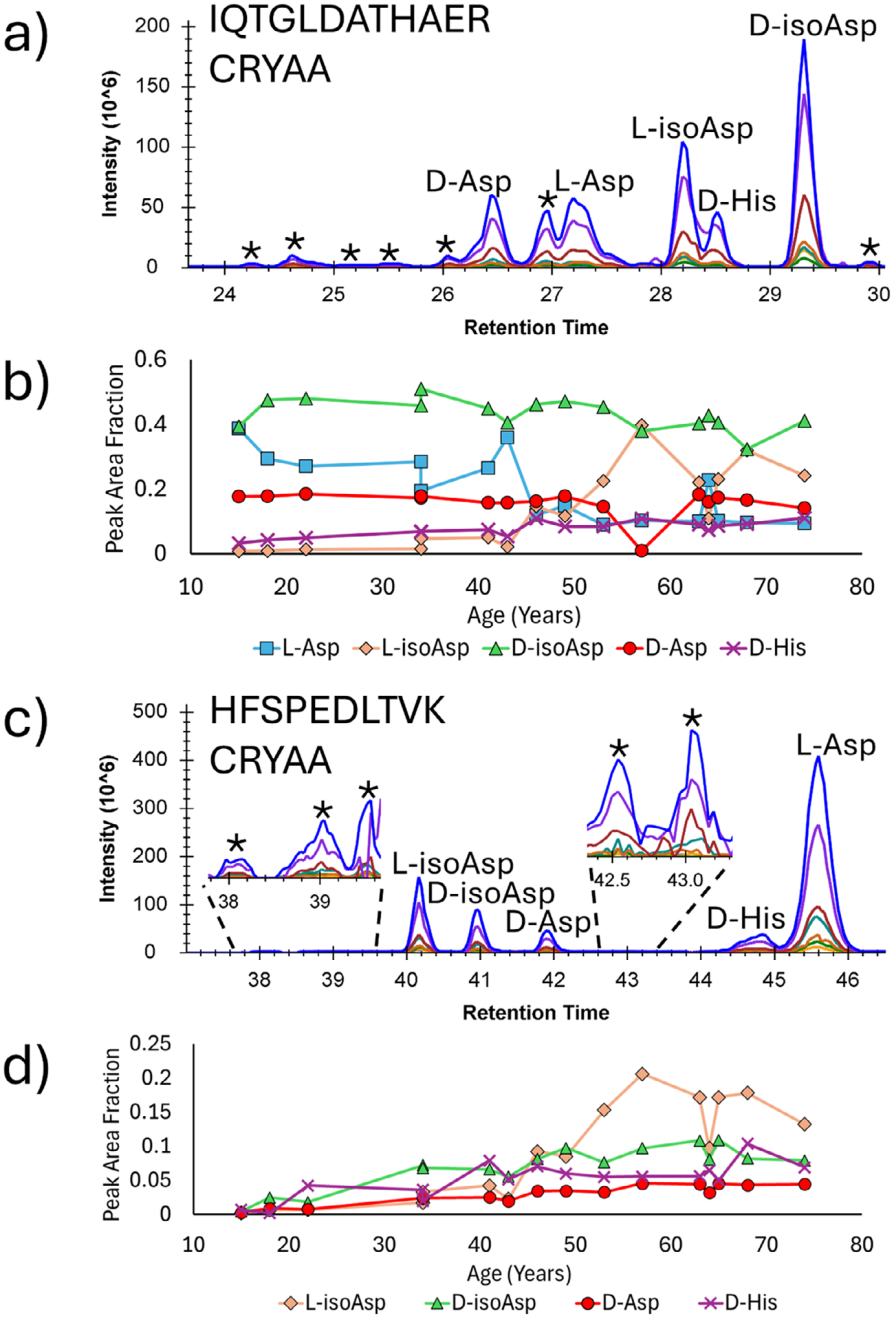
Isomerization in the inner nucleus for two peptides, IQTGLDATHAER and HFSPEDLTVK, both from αA‐crystallin, in all 16 lens samples. (a) Chromatogram of IQTGLDATHAER in 74 y.o. lens with identified peaks labeled, including confirmed D‐His isomerization. Asterisks denote unidentified peaks. (b) The peak area fraction for each identified IQTGLDATHAER isomer plotted across all lens samples. (c) Labeled Chromatogram for HFSPEDLTVK in 74 y.o. lens, including confirmed D‐His isomerization. (d) The peak area fraction for each identified HFSPEDLTVK isomer plotted across all lens samples.

Although a depletion strategy was employed to reduce their overwhelming abundance, sufficient quantities of the crystallin proteins remained in the sample to allow for high‐quality data to be obtained. As stated above, an advantage of DIA is the ability to capture information about all isomers. This capability is particularly important for peptides such as IQTGLDATHAER from αA‐crystallin, which is highly isomerized and exists in at least 12 different isomeric forms (Figure 5a). This peptide would be expected to isomerize at Asp and Glu, but comparison with all Asp/Glu synthetic standards could not account for all of the abundant peaks. Exploration of dozens of synthetic standards eventually yielded a retention time and fragment‐ion intensity match to a D‐His isomer. A comparison of areas for all identified peaks, shown in Figure 5b, reveals how these known isomers progress with increasing age. Interestingly, the fractional abundance of D‐His is competitive with and eventually surpasses some of the Asp isomers. Although His isomerization has not been previously documented, there must be a mechanism that can produce significant isomerization in certain peptides. Similar efforts using synthetic standards uncovered another site of His isomerization in the αA‐crystallin peptide HFSPEDLTVK, which exhibits 10 isomeric forms (although some are present in trace amounts, Figure 5c). Peak area analysis of identified isomers in Figure 5d again shows that the D‐His peak area remains comparable to Asp isomer peaks across all ages and is consistently greater than the D‐Asp area. Despite careful examination, no other His isomers were detected in our data, prompting the question as to why isomerization occurs at these sites. Interestingly, both His residues in these peptides have been reported to bind transition metals (Liao et al. 2013; Laganowsky et al. 2010; Karmakar and Das 2012). Although more work is necessary to fully explore the underlying mechanism, a tempting hypothesis would be that metal binding facilitated the accelerated isomerization in these peptides.

As a spontaneous chemical process, Asp isomerization within any given peptide will accumulate consistently as a function of time if incubated under identical conditions. Given that proteins in the inner nucleus are the same age as the person, isomerization is a reasonable candidate for measuring age. Indeed, the potential to use isomerization as a means for age determination was recognized when isomerized Asp was first identified within the lens (Masters, Bada, and Samuel Zigler 1977). However, other tissues, primarily dentin from teeth, are preferred as the analyte for forensic aging via Asp isomerization (Ohtani and Yamamoto 2024). Although teeth are more durable, using dentin requires harsh processing, including tooth pulverization, demineralization, and high temperature acid hydrolysis of all protein. These treatments can induce artificial isomerization of Asp and complicate the interpretation of the results (Ritz‐Timme et al. 2000). High temperature acid hydrolysis of lens proteins suffers the same drawbacks and condenses the varying extents of isomerization from all proteins into a single measurable. Although this is convenient, it is feasible that measuring multiple parameters with minimal artefactual isomerization might enable more accurate prediction of age. Sample preparation for bottom‐up DIA of lens tissue does not employ conditions that would meaningfully contribute to Asp isomerization, and isomerization can be independently explored at hundreds of different sites (Li et al. 2008; Ren et al. 2009).

**FIGURE 6:**
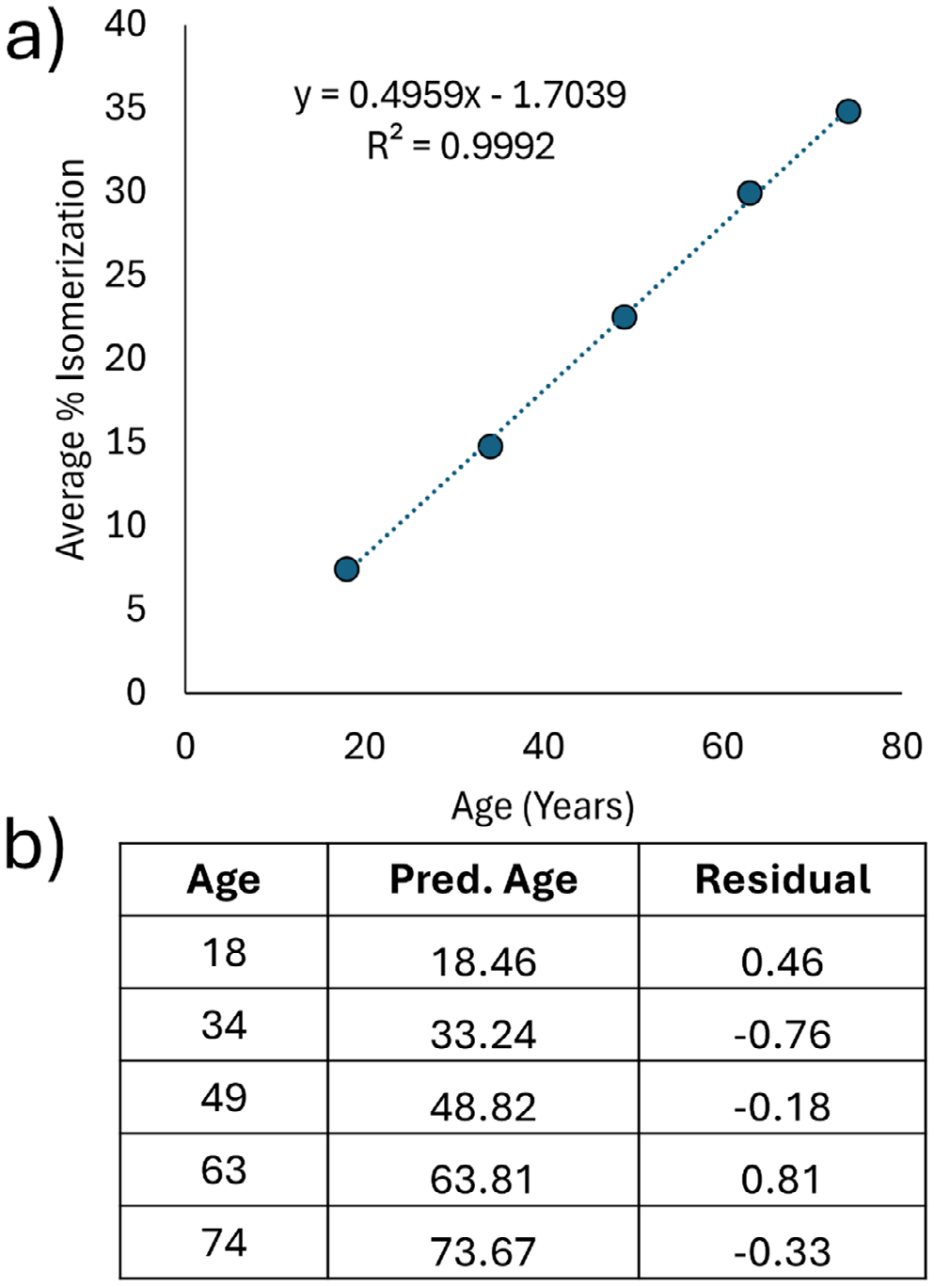
Predicting age with isomerization. (a) Comparison of the averaged % isomerization from 15 noncrystallin lens isomers to lens age. (b) Predicted ages and their residuals based on the linear fit of averaged % isomerization.

A prediction of age for five lens tissue samples, using % isomerization values from the inner nucleus, is shown in Figure 6a. These predictions are not made using a single peptide, but rather by collective analysis of 14 peptides (Table S1). These peptides were selected for the resolution of all four Asp isomers and a highly linear relationship between % isomerization and age. By aggregating the predictions of many isomers together, the merged prediction becomes more robust. Residuals for age prediction, shown in Figure 6b, show that all predicted ages are within 0.81 years of actual age, based on an extremely linear fit. These results are comparable or arguably superior to those obtained with dentin (Ohtani and Yamamoto 2010; Roy, Jayaraman, and Johnson 2022).

## 4 Conclusion

DIA chromatographic data has recently emerged as one of the most effective methods to identify Asp isomerization sites within proteomes. By analyzing DIA of crystallin‐depleted lens protein, we have found hundreds of unreported Asp isomerization sites within the lens proteome. With many isomers available across many proteins, sequence motifs, and secondary structures, we developed a better understanding of the variables that dictate isomerization rates on long timescales. Quantification of newly discovered serine and histidine isomerization revealed new insights into the nature of both processes in lens tissue. Combining the isomerization rates of many peptides allowed us to determine the age of lens tissue with an accuracy that matches the best current forensic techniques. This has potential applications for forensic age determination by DIA or selected reaction monitoring of known isomerization sites (Picotti and Aebersold 2012). This is the first published use of DIA‐based, whole‐proteome isomer detection. The location of hundreds of isomer sites from a single dataset is unprecedented, and similar analysis might be usefully applied to other low‐turnover tissues and to diseases that disrupt proteostasis.

## Author Contributions

Project conceived by R.R.J., and E.E.H., L.S.C., and K.L.S. performed LC – MS of lens tissue. T.R.L. identified histidine isomers. M.H. built the automated isomer quantification tool with consultation from R.R.J., and E.E.H. Analysis and interpretation of data were performed by E.E.H., T.A.S., R.R.J., T.R.L., E.K., B.D.P., M.K.L., and J.R.L. Peptide synthesis was performed by T.A.S., E.E.H., and C.C., T.A.S., and E.E.H. performed LC – MS experiments of synthetic peptides. Manuscript written by E.E.H., T.A.S., R.R.J., and M.H. with consultation from K.L.S., L.S.C., and T.R.L.

## Conflicts of Interest

The authors declare no conflicts of interest.

## Supporting information


**Appendix** **S1**.List of all confidently identified isomerization sites and corresponding quantitative information (Table S1). LC–MS analysis of synthesized standards for the chromatographically unresolved isomers of GDLGIEIPAEK and VLPWADR (Figure S1). Glutamic acid isomerization mechanism and selected glutamic acid isomer chromatograms (Figure S2).

## Data Availability

All raw MS data files are available at ProteomeXchange dataset entry PXD033722.
