# Supplementary material for: Deep Characterization of Isomerization in the Human Eye Lens Proteome by Crystallin‐Depleted Data‐Independent Acquisition
Source: Aging Cell. 2025 May 1;24(6):e70028. doi: 10.1111/acel.70028 (PMC12151897; doi:10.1111/acel.70028)
Supplement: Supplementary file 1 — Appendix S1.List of all confidently identified isomerization sites and corresponding quantitative information (Table S1). LC–MS analysis of synthesized standards for the chromatographically unresolved isomers of GDLGIEIPAEK and VLPWADR (Figure S1). Glutamic acid isomerization mechanism and selected glutamic acid isomer chromatograms (Figure S2). [file ACEL-24-e70028-s001.docx]

Table S1. All peptides found to undergo Asp and Ser isomerization. * denotes peptides used for age prediction. ^ denotes peptides containing known His isomerization sites.

| Non-Crystallin Asp Isomers | | | | | | | |
| --- | --- | --- | --- | --- | --- | --- | --- |
| Peptide | Protein | All Peaks Resolved? | % Iso 18 y.o. | % Iso 34 y.o. | % Iso 49 y.o. | % Iso 63 y.o. | % Iso 74 y.o. |
| GDIVFFLQK* | sp\|Q86TP1\|PRUN1_HUMAN | Y | 0.6 | 1.8 | 4.6 | 8.8 | 11.2 |
| VICDTFTVTGEPLLTSPR* | sp\|Q5TDP6\|LGSN_HUMAN | Y | 0.8 | 5.3 | 17.9 | 22.1 | 32.1 |
| FEATDLHGVSR* | sp\|Q5TDP6\|LGSN_HUMAN | Y | 1.4 | 7.3 | 13.8 | 26 | 27.3 |
| VLTSDIEK* | sp\|P48163\|MAOX_HUMAN | Y | 1.6 | 6.7 | 12.6 | 21.3 | 24.8 |
| TMDASER* | sp\|P00352\|AL1A1_HUMAN | Y | 4.1 | 19.2 | 25.9 | 28 | 35.8 |
| ILDLIESGK* | sp\|P00352\|AL1A1_HUMAN | Y | 6.5 | 28 | 36 | 44.8 | 52.5 |
| TFEDISEK* | sp\|P48637\|GSHB_HUMAN | Y | 9.8 | 16.1 | 22 | 32.9 | 35.3 |
| LGELAGPEDALAR* | sp\|Q12934\|BFSP1_HUMAN | Y | 13 | 30.4 | 46.7 | 65.6 | 75 |
| IADGYEQAAR* | sp\|P48643\|TCPE_HUMAN | Y | 54.4 | 60.1 | 61.7 | 69.1 | 71.5 |
| LADLIER | sp\|P00352\|AL1A1_HUMAN | Y | 0.4 | 0.9 | 1.8 | 4.2 | 3.6 |
| IFINNEWHDSVSGK | sp\|P00352\|AL1A1_HUMAN | Y | 6.7 | 24 | 35.8 | 30.9 | 27 |
| ALMDEVVK | sp\|P00558\|PGK1_HUMAN | Y | 5.2 | 10 | 16.6 | 28.3 | 16.2 |
| SLLGKDVLFLK | sp\|P00558\|PGK1_HUMAN | Y | 7.3 | 37.4 | 78.7 | 81.1 | 82.4 |
| ITLPVDFVTADK | sp\|P00558\|PGK1_HUMAN | Y | 0 | 33.6 | 47.4 | 63.9 | 62.6 |
| GLAGLGDVAEVR | sp\|P11216\|PYGB_HUMAN | Y | 3.3 | 7.4 | 6.1 | 6.7 | 20.4 |
| LEDFFAR | sp\|P11413\|G6PD_HUMAN | Y | 10.8 | 28.4 | 39 | 29.9 | 47.1 |
| GGYFDEFGIIR | sp\|P11413\|G6PD_HUMAN | Y | 28.6 | 44.3 | 61 | 66.8 | 69.3 |
| LFSGDVVLTAR | sp\|P16152\|CBR1_HUMAN | Y | 19.8 | 33.1 | 29.9 | 39.8 | 40.8 |
| TDEGIAYR | sp\|P32119\|PRDX2_HUMAN | Y | 14.3 | 25.1 | 30.4 | 30 | 29.6 |
| ATAVVDGAFK | sp\|P32119\|PRDX2_HUMAN | Y | 61.8 | 77.5 | 82.2 | 82.4 | 90.1 |
| AIVVTDGER | sp\|P48163\|MAOX_HUMAN | Y | 46.4 | 61 | 67.1 | 68.6 | 70.1 |
| ALHSDFIVK | sp\|P52333\|JAK3_HUMAN | Y | 2.6 | 7.1 | 14.5 | 12.2 | 14.5 |
| LLDSSTVTHLFK | sp\|P60900\|PSA6_HUMAN | Y | 39.5 | 55.9 | 65.5 | 66.6 | 85.2 |
| LVLVGDGGTGK | sp\|P62826\|RAN_HUMAN | Y | 47.9 | 58.8 | 66.7 | 71.8 | 68.2 |
| TEWLDGK | sp\|P62937\|PPIA_HUMAN | Y | 0 | 27.5 | 45.6 | 46.1 | 54.7 |
| CVVVGDGAVGK | sp\|P63000\|RAC1_HUMAN | Y | 0.8 | 1.8 | 18.5 | 37.4 | 36.5 |
| YYVTIIDAPGHR | sp\|P68104\|EF1A1_HUMAN / sp\|Q5VTE0\|EF1A3_HUMAN | Y | 18.9 | 26.8 | 35.1 | 36.1 | 32.2 |
| MLAEDELR | sp\|P84077\|ARF1_HUMAN / sp\|P61204\|ARF3_HUMAN | Y | 0 | 12.4 | 26.5 | 30.3 | 27 |
| EIGADLVLQISK | sp\|Q00796\|DHSO_HUMAN | Y | 7.1 | 19.7 | 53.8 | 57.1 | 57.2 |
| HLLGVEDLLQK | sp\|Q01082\|SPTB2_HUMAN | Y | 4.2 | 12.8 | 34.3 | 41.3 | 32 |
| GFDEYMK | sp\|Q01469\|FABP5_HUMAN | Y | 0 | 7.6 | 20.8 | 31.5 | 21.4 |
| FEETTADGR | sp\|Q01469\|FABP5_HUMAN | Y | 24.8 | 40.1 | 48.4 | 57.4 | 58.5 |
| VITDLSSGI | sp\|Q11203\|SIAT6_HUMAN | Y | 17.3 | 21.3 | 27.3 | 39 | 43.1 |
| VRDLEAER | sp\|Q12934\|BFSP1_HUMAN | Y | 2.4 | 12.4 | 33.2 | 52.3 | 64.7 |
| FTTGDAMSK | sp\|Q14019\|COTL1_HUMAN | Y | 73.9 | 84.9 | 82.3 | 80.6 | 79.5 |
| EIIDLVLDR | sp\|Q71U36\|TBA1A_HUMAN | Y | 1.4 | 2 | 3.2 | 4.9 | 6.7 |
| STAGALDKK | sp\|Q8TDW0\|LRC8C_HUMAN | Y | 0.3 | 23.7 | 49.9 | 54.8 | 53.3 |
| DGLILTSR | sp\|Q99497\|PARK7_HUMAN | Y | 0 | 4.6 | 26.1 | 30.6 | 33.8 |
| ILVDVEK | sp\|P11216\|PYGB_HUMAN | N | 0 | 0 | 9.9 | 24.2 | 21.8 |
| SLLDACESR | sp\|Q01082\|SPTB2_HUMAN | N | 0 | 0 | 2.4 | 6.6 | 3.4 |
| ALCAEADR | sp\|Q13813\|SPTN1_HUMAN | N | 0 | 0 | 1.6 | 4.7 | 5.4 |
| SVPTSTVFYPSDGVATEK | sp\|P29401\|TKT_HUMAN | N | 0 | 0 | 2.5 | 4.1 | 5.5 |
| LIFTDGSR | sp\|P36871\|PGM1_HUMAN | N | 1.8 | 3.8 | 8.1 | 10.6 | 15.5 |
| DYGDHLSR | sp\|O75526\|RMXL2_HUMAN | N | 63.3 | 67 | 73 | 76 | 77.7 |
| YDEMVESMK | sp\|P62258\|1433E_HUMAN | N | 0 | 0 | 11.9 | 31.7 | 37.8 |
| ILLLGLDNAGK | sp\|P36405\|ARL3_HUMAN | N | 0 | 0 | 11.7 | 10.4 | 24 |
| EDFSEATLK | sp\|Q53FA7\|QORX_HUMAN | N | 0 | 20 | 33.5 | 47.1 | 56.2 |
| LLPVLDR | sp\|Q53FA7\|QORX_HUMAN | N | 0 | 19.6 | 35.3 | 57.8 | 56.6 |
| VIILGDSGVGK | sp\|P51149\|RAB7A_HUMAN | N | 0 | 6.5 | 28.1 | 42.6 | 61.1 |
| LYTLVLTDPDAPSR | sp\|P30086\|PEBP1_HUMAN | N | 0 | 2.2 | 10.7 | 14.2 | 16.3 |
| VLPGVDALSNI | sp\|P00558\|PGK1_HUMAN | N | 0 | 15.8 | 26.7 | 45.6 | 22 |
| YADLTEDQLPSCESLK | sp\|P18669\|PGAM1_HUMAN | N | 0 | 16.9 | 32.5 | 43.4 | 35.4 |
| VDLGVLGK | sp\|P36871\|PGM1_HUMAN | N | 0 | 23.8 | 28.4 | 21.6 | 28.8 |
| VAVVAGYGDVGK | sp\|P23526\|SAHH_HUMAN | N | 0 | 9.4 | 17.9 | 23.7 | 25.7 |
| ITDLYTDLR | sp\|Q01082\|SPTB2_HUMAN | N | 0 | 2.2 | 7 | 8.9 | 7.3 |
| IFAVEILDK | sp\|Q8NHU6\|TDRD7_HUMAN | N | 0 | 18.9 | 44.9 | 42.1 | 30.8 |
| DTAAWTVGR | sp\|Q14974\|IMB1_HUMAN | N | 0 | 5.9 | 13.3 | 22.7 | 25.5 |
| SDYMFQR | sp\|P48637\|GSHB_HUMAN | N | 0 | 0 | 3.7 | 11.8 | 14.7 |
| MGAMAKPDCIITCDGK | sp\|Q01469\|FABP5_HUMAN | N | 0 | 0.3 | 33.7 | 31.1 | 39 |
| VSFELFADK | sp\|P62937\|PPIA_HUMAN | N | 0 | 0 | 18.6 | 44.7 | 48.4 |
| GLFIIDGK | sp\|P32119\|PRDX2_HUMAN | N | 0 | 8.6 | 8.7 | 15 | 12.2 |
| IIFSDASR | sp\|Q15124\|PGM5_HUMAN | N | 0 | 4.2 | 9.3 | 11.1 | 16 |
| GLFITIHDR | sp\|P48163\|MAOX_HUMAN | N | 0 | 18.8 | 39.6 | 35.6 | 40.6 |
| EADEALLHNLR | sp\|Q12934\|BFSP1_HUMAN | N | 0.6 | 3.3 | 8.5 | 18.8 | 22.1 |
| VQLVVGDGR | sp\|P22061\|PIMT_HUMAN | N | 1.4 | 14 | 29 | 42.7 | 49.5 |
| YCAGWADK | sp\|P00352\|AL1A1_HUMAN | N | 1.6 | 9.1 | 19 | 25.6 | 28.7 |
| GCITIIGGGDTATCCAK | sp\|P00558\|PGK1_HUMAN | N | 2.1 | 9 | 25.3 | 34.1 | 0 |
| VLPWADR | sp\|Q5TDP6\|LGSN_HUMAN | N | 2.8 | 19.5 | 34.9 | 51.9 | 61.9 |
| GLVSSDELAK | sp\|Q13813\|SPTN1_HUMAN | N | 3.3 | 12.7 | 32.7 | 41.4 | 44.3 |
| DLTDYLMK | sp\|P60709\|ACTB_HUMAN /sp\|Q01082\|SPTB2_HUMAN /sp\|P63261\|ACTG_HUMAN | N | 4.1 | 10.2 | 22.1 | 23.1 | 32.9 |
| ILMVGLDAAGK | sp\|P84077\|ARF1_HUMAN / sp\|P61204\|ARF3_HUMAN | N | 5.6 | 10 | 11.9 | 17.5 | 19.4 |
| DLGTESQIFISR | sp\|P50395\|GDIB_HUMAN | N | 7.1 | 46.1 | 70.6 | 69.9 | 81.5 |
| DYFFALAHTVR | sp\|P11216\|PYGB_HUMAN | N | 7.1 | 14.8 | 16 | 24.3 | 24.3 |
| LAGGDWFTSR | sp\|P30153\|2AAA_HUMAN | N | 7.5 | 15 | 13.6 | 24.9 | 22.7 |
| AAYDVSSFSFFQR | sp\|O15498\|YKT6_HUMAN | N | 7.9 | 19.5 | 35.4 | 40 | 0 |
| EYEEDGAR | sp\|P61163\|ACTZ_HUMAN | N | 8.5 | 20.1 | 32.6 | 43.5 | 0 |
| LGEYEDVSR | sp\|Q99426\|TBCB_HUMAN | N | 9.4 | 16.6 | 13.4 | 19.8 | 24.7 |
| VVFVFGPDK | sp\|P30041\|PRDX6_HUMAN | N | 12.7 | 26.6 | 32.3 | 41.7 | 40.2 |
| ELDESLQVAER | sp\|P10909\|CLUS_HUMAN | N | 13.1 | 24 | 32.3 | 51 | 56.1 |
| ILSSDDYGK | sp\|Q01082\|SPTB2_HUMAN | N | 14 | 26.6 | 47.7 | 60.3 | 65.8 |
| EFVISDR | sp\|Q14019\|COTL1_HUMAN | N | 14.2 | 23.2 | 35.7 | 51.8 | 48.3 |
| FYEEVHDLER | sp\|P55209\|NP1L1_HUMAN | N | 14.4 | 21.9 | 22.1 | 33.8 | 26.9 |
| TIPIDGNFFTYTR | sp\|P00352\|AL1A1_HUMAN | N | 20.3 | 44.5 | 52.6 | 39.4 | 23.8 |
| TASDMVSTSR | sp\|O00232\|PSD12_HUMAN | N | 23 | 31.6 | 32.8 | 37.8 | 32.5 |
| GGPLDGTYR | sp\|P00918\|CAH2_HUMAN | N | 29.2 | 33.5 | 39.6 | 40 | 45.1 |
| AGFAGDDAPR | sp\|P60709\|ACTB_HUMAN / sp\|P63261\|ACTG_HUMAN | N | 32.8 | 39.9 | 45.4 | 59.7 | 60.4 |
| GDLGIEIPAEK | sp\|\|KPYMP14618_HUMAN | N | 33.3 | 45.6 | 55.9 | 65 | 66.8 |
| ADDGRPFPQVIK | sp\|P04075\|ALDOA_HUMAN | N | 49.7 | 74.9 | 77.9 | 82 | 82.7 |
| LTSTDTIPK | sp\|P11171\|41_HUMAN | N | 64.3 | 75 | 80.5 | 83.9 | 86.3 |
| LLPLVSDEVFIR | sp\|P11216\|PYGB_HUMAN | N | 50.6 | 58 | 51.7 | 75.8 | 58.4 |
| GAAVDFTAR | sp\|Q01484\|ANK2_HUMAN |  |  |  |  |  |  |
| SGHDQVVELLLER | sp\|Q01484\|ANK2_HUMAN |  |  |  |  |  |  |
| FWLIDCR | sp\|Q01484\|ANK2_HUMAN |  |  |  |  |  |  |
| GPLLGITDK | sp\|Q8IW19\|APLF_HUMAN |  |  |  |  |  |  |
| IFVEESIYDEFVR | sp\|P00352\|AL1A1_HUMAN |  |  |  |  |  |  |
| GILAADESTGSIAK | sp\|P04075\|ALDOA_HUMAN |  |  |  |  |  |  |
| QLLLTADDR | sp\|P04075\|ALDOA_HUMAN |  |  |  |  |  |  |
| DSYVGDEAQSK | sp\|P60709\|ACTB_HUMAN / sp\|P63261\|ACTG_HUMAN |  |  |  |  |  |  |
| LDLAGRDLTDYLMK | sp\|P60709\|ACTB_HUMAN / sp\|P63261\|ACTG_HUMAN |  |  |  |  |  |  |
| KDLYANTVLSGGTTMYPGIADR | sp\|P60709\|ACTB_HUMAN / sp\|P63261\|ACTG_HUMAN |  |  |  |  |  |  |
| VAGMDVELTVEER | sp\|P62258\|1433E_HUMAN |  |  |  |  |  |  |
| IVITGDADIDHDQVLVQAIK | sp\|P11171\|41_HUMAN |  |  |  |  |  |  |
| VVVHQETEIADE | sp\|P11171\|41_HUMAN |  |  |  |  |  |  |
| LPSGSGAASPTGSAVDIR | sp\|Q09666\|AHNK_HUMAN |  |  |  |  |  |  |
| GILAADESVGSMAK | sp\|P09972\|ALDOC_HUMAN |  |  |  |  |  |  |
| EAVTQTDAEAGK | sp\|Q5H9R4\|ARMX4_HUMAN |  |  |  |  |  |  |
| DAEWTTVFK | sp\|O00192\|ARVC_HUMAN |  |  |  |  |  |  |
| VTTVASHTSDSDVPSGVTEVVVK | sp\|P10909\|CLUS_HUMAN |  |  |  |  |  |  |
| LPDESFR | sp\|Q01814\|AT2B2_HUMAN |  |  |  |  |  |  |
| HLDHVAALFPGDVDR | sp\|P11216\|PYGB_HUMAN |  |  |  |  |  |  |
| VEDVEALDR | sp\|P11216\|PYGB_HUMAN |  |  |  |  |  |  |
| EYYDHLPELK | sp\|P11216\|PYGB_HUMAN |  |  |  |  |  |  |
| VYEDETK | sp\|O75781\|PALM_HUMAN |  |  |  |  |  |  |
| GLFIIDDK | sp\|Q06830\|PRDX1_HUMAN |  |  |  |  |  |  |
| DFTPVCTTELGR | sp\|P30041\|PRDX6_HUMAN |  |  |  |  |  |  |
| LPFPIIDDR | sp\|P30041\|PRDX6_HUMAN |  |  |  |  |  |  |
| LPFPIIDDRNR | sp\|P30041\|PRDX6_HUMAN |  |  |  |  |  |  |
| VATPVDWK | sp\|P30041\|PRDX6_HUMAN |  |  |  |  |  |  |
| LWDLTTGTTTR | sp\|P63244\|RACK1_HUMAN |  |  |  |  |  |  |
| AFMDLTLMASWNQR | sp\|Q8TDX9\|PK1L1_HUMAN |  |  |  |  |  |  |
| TITLEVEPSDTIENVK | sp\|P62979\|RS27A_HUMAN / sp\|P0CG48\|UBC_HUMAN / sp\|P62987\|RL40_HUMAN / sp\|P0CG47\|UBB_HUMAN |  |  |  |  |  |  |
| ESILDGLK | sp\|O43865\|SAHH2_HUMAN |  |  |  |  |  |  |
| VSTEVDAR | sp\|P37837\|TALDO_HUMAN |  |  |  |  |  |  |
| RYSDFER | sp\|Q969T3\|SNX21_HUMAN |  |  |  |  |  |  |
| AMVALIDVFHQYSGR | sp\|P04271\|S100B_HUMAN |  |  |  |  |  |  |
| IQTSGEPDVAER | sp\|Q8NEM2\|SHCBP_HUMAN |  |  |  |  |  |  |
| DGMAFNALIHK | sp\|Q01082\|SPTB2_HUMAN |  |  |  |  |  |  |
| VLVLSQDYGK | sp\|Q01082\|SPTB2_HUMAN |  |  |  |  |  |  |
| AFEDEMSGR | sp\|Q01082\|SPTB2_HUMAN |  |  |  |  |  |  |
| ALVADSHPESER | sp\|Q01082\|SPTB2_HUMAN |  |  |  |  |  |  |
| DAEELEK | sp\|Q13813\|SPTN1_HUMAN |  |  |  |  |  |  |
| VEDLFLTFAK | sp\|Q13813\|SPTN1_HUMAN |  |  |  |  |  |  |
| LYSILGTTLKDEGK | sp\|O75083\|WDR1_HUMAN |  |  |  |  |  |  |
| LSVDYGKK | sp\|Q71U36\|TBA1A_HUMAN |  |  |  |  |  |  |
| LGFEDGSVLK | sp\|P09936\|UCHL1_HUMAN |  |  |  |  |  |  |
| SDDGVAVICR | sp\|Q8N3J6\|CADM2_HUMAN |  |  |  |  |  |  |
| ISTGPLGDLSR | sp\|P48165\|CXA8_HUMAN |  |  |  |  |  |  |
| DYAVSTVPVADGLHLK | sp\|O94760\|DDAH1_HUMAN |  |  |  |  |  |  |
| YLLCQADVK | sp\|P29275\|AA2BR_HUMAN |  |  |  |  |  |  |
| LDLAGRDLTDYLMK | sp\|P68032\|ACTC_HUMAN |  |  |  |  |  |  |
| EIVRDIK | sp\|P68032\|ACTC_HUMAN |  |  |  |  |  |  |
| EQYEHADEASR | sp\|Q12934\|BFSP1_HUMAN |  |  |  |  |  |  |
| DVASYHALLDR | sp\|Q13515\|BFSP2_HUMAN |  |  |  |  |  |  |
| TDDYLDQPCLETVNR | sp\|P31150\|GDIA_HUMAN |  |  |  |  |  |  |
| FVSISDLLVPK | sp\|P50395\|GDIB_HUMAN |  |  |  |  |  |  |
| MDEHGFISR | sp\|Q12988\|HSPB3_HUMAN |  |  |  |  |  |  |
| ALWEDEGVR | sp\|Q5JWF2\|GNAS1_HUMAN / sp\|P63092\|GNAS2_HUMAN |  |  |  |  |  |  |
| SLVEIADTVPK | sp\|O00410\|IPO5_HUMAN |  |  |  |  |  |  |
| VSDILHSIFSSYK | sp\|O00410\|IPO5_HUMAN |  |  |  |  |  |  |
| LVEDEER | sp\|Q14204\|DYHC1_HUMAN |  |  |  |  |  |  |
| SPEDLER | sp\|P06744\|G6PI_HUMAN |  |  |  |  |  |  |
| IEEELGDEAR | sp\|P09104\|ENOG_HUMAN |  |  |  |  |  |  |
| ELEEDFIK | sp\|Q14019\|COTL1_HUMAN |  |  |  |  |  |  |
| VLTEDEMGHPEIGDAIAR | sp\|P14324\|FPPS_HUMAN |  |  |  |  |  |  |
| FVFLLDR | sp\|Q13045\|FLII_HUMAN |  |  |  |  |  |  |
| EDQTEYLEER | sp\|P07900\|HS90A_HUMAN |  |  |  |  |  |  |
| IEKFDR | sp\|Q9UKE5\|TNIK_HUMAN |  |  |  |  |  |  |
| DLSELGSVR | sp\|Q15149\|PLEC_HUMAN |  |  |  |  |  |  |
| EDYLYAVR | sp\|O95298\|NDUC2_HUMAN |  |  |  |  |  |  |
| IQGDLAGR | sp\|Q9BX67\|JAM3_HUMAN |  |  |  |  |  |  |
| AYTPVSSDDDR | sp\|Q6BCY4\|NB5R2_HUMAN |  |  |  |  |  |  |
| IDTVGTDVR | sp\|Q99972\|MYOC_HUMAN |  |  |  |  |  |  |
| GDAEIKER | sp\|Q8TDR0\|MIPT3_HUMAN |  |  |  |  |  |  |
| Crystallin Asp Isomers | | | | | | | |
| Peptide | Protein |  | % Iso 18yo | % Iso 34yo | % Iso 49yo | %iso 63yo | % Iso 74yo |
| GYQYLLEPGDFR* | sp\|P53674\|CRBB1_HUMAN | Y | 1.9 | 7.9 | 22.9 | 35.2 | 44.9 |
| CQDWGAMDAK* | sp\|P07315\|CRGC_HUMAN | Y | 2.7 | 5.6 | 12.2 | 16.9 | 18.8 |
| MEFTSSCPNVSERSFDNVR* | sp\|P05813\|CRBA1_HUMAN | Y | 3.9 | 10.1 | 20.8 | 21.8 | 25.5 |
| AEFSGECSNLADR* | sp\|P53674\|CRBB1_HUMAN | Y | 1.1 | 5.5 | 14 | 20.2 | 22.9 |
| MDAQEHK | sp\|P53674\|CRBB1_HUMAN | Y | 2 | 7.6 | 12.2 | 12.3 | 22.8 |
| HFSPEDLTVK^ | sp\|P02489\|CRYAA_HUMAN | Y | 4.4 | 14.9 | 28.1 | 38.2 | 33.5 |
| QYLLRPGDYR | sp\|P11844\|CRGA_HUMAN | Y | 24.3 | 45.5 | 65.9 | 76.4 | 82.8 |
| IQTGLDATHAER^ | sp\|P02489\|CRYAA_HUMAN | Y | 45.5 | 44.1 | 48.4 | 62 | 66.6 |
| VLGDVIEVHGK | sp\|P02511\|CRYAB_HUMAN | Y | 0.7 | 2 | 5.1 | 10.6 | 13.1 |
| YQDWGATNAR | sp\|P07320\|CRGD_HUMAN | Y | 0.9 | 1.2 | 1.4 | 1.2 | 3.1 |
| DMQWHQR | sp\|P43320\|CRBB2_HUMAN | Y | 2.5 | 3 | 3.8 | 6.5 | 10 |
| ITFYEDR | sp\|P07315\|CRGC_HUMAN | N | 0 | 1 | 2 | 4.1 | 5.4 |
| YHDWGGADAK | sp\|P11844\|CRGA_HUMAN | N | 54.5 | 74 | 86.2 | 89.8 | 62 |
| WDAWSGSNAYHIER | sp\|P05813\|CRBA1_HUMAN | N | 1.3 | 4.9 | 7.4 | 8.1 | 21.4 |
| IYDRDELR | sp\|P07316\|CRGB_HUMAN | N | 0 | 1.8 | 4.4 | 25.2 | 25.1 |
| DKFVIFLDVK | sp\|P02489\|CRYAA_HUMAN | N | 4.8 | 5.3 | 7.5 | 11.6 | 9.2 |
| MDVTIQHPWFK | sp\|P02489\|CRYAA_HUMAN | N | 19.4 | 26.6 | 33.1 | 46.5 | 55.4 |
| ITFYEDRDFQGR | sp\|P11844\|CRGA_HUMAN | N | 8.7 | 12.6 | 59 | 59.6 | 52.7 |
| TVLDSGISEVR | sp\|P02489\|CRYAA_HUMAN |  |  |  |  |  |  |
| VQDDFVEIHGK | sp\|P02489\|CRYAA_HUMAN |  |  |  |  |  |  |
| MDIAIHHPWIR | sp\|P02511\|CRYAB_HUMAN |  |  |  |  |  |  |
| APSWFDTGLSEMR | sp\|P02511\|CRYAB_HUMAN |  |  |  |  |  |  |
| FSVNLDVK | sp\|P02511\|CRYAB_HUMAN |  |  |  |  |  |  |
| HEERQDEHGFISR | sp\|P02511\|CRYAB_HUMAN |  |  |  |  |  |  |
| QDEHGFISR | sp\|P02511\|CRYAB_HUMAN |  |  |  |  |  |  |
| GYQYILECDHHGGDYK | sp\|P05813\|CRBA1_HUMAN |  |  |  |  |  |  |
| ITIYDQENFQGK | sp\|P05813\|CRBA1_HUMAN |  |  |  |  |  |  |
| GEYPDYQQWMGLSDSIR | sp\|P07315\|CRGC_HUMAN |  |  |  |  |  |  |
| EDHKGLMMELSEDCPSIQDR | sp\|P07315\|CRGC_HUMAN |  |  |  |  |  |  |
| LYERDDYR | sp\|P11844\|CRGA_HUMAN |  |  |  |  |  |  |
| WMGLNDR | sp\|P22914\|CRYGS_HUMAN |  |  |  |  |  |  |
| QYLLDKK | sp\|P22914\|CRYGS_HUMAN |  |  |  |  |  |  |
| ITFYEDK | sp\|P22914\|CRYGS_HUMAN |  |  |  |  |  |  |
| WDSWTSSR | sp\|P43320\|CRBB2_HUMAN |  |  |  |  |  |  |
| TDSLSSLRPIK | sp\|P43320\|CRBB2_HUMAN |  |  |  |  |  |  |
| MEIIDDDVPSFHAHGYQEK | sp\|P43320\|CRBB2_HUMAN |  |  |  |  |  |  |
| GDYKDSSDFGAPHPQVQSVR | sp\|P43320\|CRBB2_HUMAN |  |  |  |  |  |  |
| VTLFEGDNFQGCK | sp\|P53672\|CRBA2_HUMAN |  |  |  |  |  |  |
| MVVWDEDGFQGR | sp\|P53673\|CRBA4_HUMAN |  |  |  |  |  |  |
| DKQWHLEGSFPVLATEPPK | sp\|P53674\|CRBB1_HUMAN |  |  |  |  |  |  |
| Serine Isomers | | | | | | | |
| Peptide | Protein |  | % Iso 18yo | % Iso 34yo | % Iso 49yo | %iso 63yo | % Iso 74yo |
| EEKPTSAPSS | sp\|P02489\|CRYAA_HUMAN | N | 21.9 | 32.2 | 34.9 | 44.5 | 0 |
| EITALAPSTMK | sp\|P60709\|ACTB_HUMAN / sp\|P63261\|ACTG_HUMAN | N | 13 | 22.8 | 27.3 | 29.6 | 31.3 |
| YLAEVASGEK | sp\|Q04917\|1433F_HUMAN | N | 6.5 | 10.6 | 12.6 | 14.6 | 12.8 |
| ATVVESSEK | sp\|P61981\|1433G_HUMAN | N | 2.7 | 9.4 | 12.7 | 12.5 | 17 |
| LKEVEASK | sp\|Q9UKS6\|PACN3_HUMAN | N | 1.2 | 2.9 | 8.1 | 10.2 | 0 |
| LGSSEHGQK | sp\|Q9P267\|MBD5_HUMAN | Y | 3.3 | 5.8 | 7.1 | 8.3 | 7.9 |
| VAFTGSTEVGK | sp\|P00352\|AL1A1_HUMAN | N | 1.4 | 4.3 | 7 | 8 | 11.6 |
| VVSSIEQK | sp\|P63104\|1433Z_HUMAN | N | 1.4 | 3.4 | 4 | 5.1 | 4.1 |
| GSLITSLLR | sp\|Q53FA7\|QORX_HUMAN | N | 0 | 1.2 | 2 | 3.5 | 1.9 |
| TLGPFYPSR | sp\|P02489\|CRYAA_HUMAN | N | 0.3 | 0.6 | 0.9 | 1.1 | 1.2 |
